# Clinical efficacy and safety of Cox-maze IV procedure for atrial fibrillation in patients with aortic valve calcification

**DOI:** 10.3389/fcvm.2023.1092068

**Published:** 2023-04-03

**Authors:** Ruikang Guo, Chengming Fan, Zhishan Sun, Hao Zhang, Yaqin Sun, Long Song, Zenan Jiang, Liming Liu

**Affiliations:** Department of Cardiovascular Surgery, The Second Xiangya Hospital, Central South University, Changsha, China

**Keywords:** calcific aortic valve disease, aortic stenosis, atrial fibrillation, surgical ablation, efficacy, safety

## Abstract

**Objective:**

Atrial fibrillation is associated with a high incidence of heart valve disease. There are few prospective clinical research comparing aortic valve replacement with and without surgical ablation for safety and effectiveness. The purpose of this study was to compare the results of aortic valve replacement with and without the Cox-maze IV procedure in patients with calcific aortic valvular disease and atrial fibrillation.

**Methods:**

We analyzed one hundred and eight patients with calcific aortic valve disease and atrial fibrillation who underwent aortic valve replacement. Patients were divided into concomitant Cox maze surgery (Cox-maze group) and no concomitant Cox-maze operation (no Cox-maze group). After surgery, freedom from atrial fibrillation recurrence and all-cause mortality were evaluated.

**Results:**

Freedom from all-cause mortality after aortic valve replacement at 1 year was 100% in the Cox-maze group and 89%, respectively, in the no Cox-maze group. No Cox-maze group had a lower rate of freedom from atrial fibrillation recurrence and arrhythmia control than those in the Cox-maze group (*P* = 0.003 and *P* = 0.012, respectively). Pre-operatively higher systolic blood pressure (hazard ratio, 1.096; 95% CI, 1.004–1.196; *P* = 0.04) and post-operatively increased right atrium diameters (hazard ratio, 1.755; 95% CI, 1.182–2.604; *P* = 0.005) were associated with atrial fibrillation recurrence.

**Conclusion:**

The Cox-maze IV surgery combined with aortic valve replacement increased mid-term survival and decreased mid-term atrial fibrillation recurrence in patients with calcific aortic valve disease and atrial fibrillation. Pre-operatively higher systolic blood pressure and post-operatively increased right atrium diameters are associated with the prediction of recurrence of atrial fibrillation.

## 1. Introduction

Atrial fibrillation (AF) is the most common serious cardiac arrhythmia in the whole world (1), it results in heart failure and left atrial thrombosis, which can lead to stroke and peripheral vascular embolism. AF can be extremely life-threatening and increases healthcare costs (2). Drug therapy and percutaneous ablation were once considered the mainstay of treatment for AF. However, the conversion rate of pharmacologic therapy and ablation in the long term may be as low as 20% (3). Cox-Maze surgery is an effective treatment for persistent AF (4, 5), but because of the invasiveness of sternotomy, this procedure is currently performed in patients who schemed concomitant cardiac surgery.

Surgical heart valve replacement is a common type of heart surgery that requires sternotomy. Surgical aortic valve replacement (SAVR) is currently the most common treatment for CAVD. SAVR requires sternotomy, and previous publications have examined the efficacy and safety of concomitant Cox Maze in patients with mitral valve disease and preoperative AF (6). However, CAVD is poorly understood by today's researchers, with no clear identification of the pathogenesis and short follow-up period analyzed (7). From a pathological perspective, heart valve disease can be classified into three pathogenesis: congenital malformations; rheumatic valve disease; and calcific aortic valve disease (CAVD) (8). At present, CAVD is the third leading cause of adult heart disease and the most common form of acquired valvular disease in western countries (9). Due to the strong association between CAVD and age, combined with the rapid aging of populations worldwide, the number of cases of CAVD will increase (10). The etiology and pathogenesis of CAVD are still unclear, and its relationship with AF remains controversial (11–15). Persistent AF in individuals with CAVD have increased risk for congestive heart failure. As AF can masks these symptoms, delayed reporting of CAVD symptoms were harmful due to later aortic valve interventions (12). However, despite the fact that certain studies have demonstrated that AF is associated with a poor prognosis in patients with severe CAVD, the apparent difference in outcomes in CAVD patients with AF or sinus rhythm (SR) can also be explained by factors other than AF (16).

The effectiveness and safety of Cox-Maze surgery combined with valve replacement have been extensively studied (7, 13, 16–18), however, these studies did not distinguish between rheumatic valve disease and CAVD in studied patients. Because of their closely spaced anatomy, there may be some potential association between the atria and the aortic valves (12). Therefore, we designed a randomized clinical trial to investigate the clinical efficacy and safety of the Cox-Maze IV procedure for AF in patients with CAVD.

## 2. Methods

This prospective randomized controlled clinical study was registered on the Chinese clinical trial website (ChiCTR1900023–775) and was approved by the ethics committee of Second Xiangya Hospital, Central South University [2019-No.054]. Patient informed consent was obtained by an independent investigator before surgery. This research was conducted in adherence to the principles of the Declaration of Helsinki.

This study was funded by the National Key Research and Development Program (2018YFC1311204).

### 2.1. Study population

Between April 2019 and September 2021, 108 consecutive patients with calcific aortic valve disease accompanied by persistent AF were randomized to the control and Cox-Maze IV groups. Inclusion criteria were as follows: (1) Patients of both sexes between the ages of 35 and 70 years; (2) Patients with a preoperative diagnosis of calcific aortic valve disease (Preoperative transthoracic echocardiographic finding of aortic valve thickening and stiffness); (3) Patients with persistent AF or long-standing persistent AF. The diagnostic criteria for persistent AF were as follows: Holter ECG monitoring on the day of surgery and seven days earlier suggested an AF rhythm without the presence of any sinus rhythm. The exclusion criteria were as follows: (1) Patients with previous open-heart surgery; (2) Patients with schemed concomitant coronary artery bypass grafting; (3) Patients with the possibility of infectious disease; (4) Patients with rheumatic valve disease; (5) Patients with congenital structural heart disease or valvular malformation. Complications have been defined according to the standardized definitions proposed by the *Society of Thoracic Surgeons/American Association for Thoracic Surgery Guidelines for Reporting Morbidity and Cardiac Valvular Operations* (19). We randomized patients using a simple randomization method. Random numbers were generated by unrelated personnel of the subject group using the RANDOM () method on a computer.

### 2.2. Cox-Maze IV operation technique

All patients underwent the Cox-Maze IV procedure combined with aortic valve replacement, and the outcome was judged by intraoperative transesophageal echocardiography (TEE). In patients in a supine position with successful general anesthesia, the chest was entered through a median chest incision, systemic anticoagulation with sodium heparin was administered, the ascending aorta and superior and inferior vena cava were cannulated, left heart drainage was placed in the right upper pulmonary vein, and the classic Cox's maze IV procedure was performed with an Atricure or Medtronic bipolar clamp. In patients with intraoperative TEE suggesting no thrombus in the left atrium, right pulmonary vein isolation was completed after extracorporeal circulation was established, vena cava was blocked, right auricular ablation was completed, the right atrium was incised, and right atrium incision to the superior vena cava, inferior vena cava, and anterior tricuspid annulus ablation line was completed. The aorta was blocked and infused with myocardial protective fluid, the isolation of the left pulmonary vein was completed after cardiac arrest, the malleolar ligament was severed and the left auricle was removed, the ablation between the left superior pulmonary vein and the left auricle, the ablation of the right superior pulmonary vein to the left superior pulmonary vein, the ablation of the right inferior pulmonary vein to the left inferior pulmonary vein and the ablation of the mitral isthmus were completed, and the left auricle was closed with continuous 5–0 Prolene sutures.

### 2.3. Aortic valve replacement operation technique

After completion of ablation, a transverse oblique aortic incision was made, and then the position of the right and left coronary artery openings was confirmed. A traction line was sewn at each of the three junctions of the aortic valve, and the three leaflets were excised separately, leaving 2 mm at the margin. The calcified tissue on the annulus was then removed and the annulus was measured to determine the prosthetic valve number. Using nylon sutures with support pads and double-ended needles, interrupted mattress sutures were sewn from top to bottom, over the annulus and immediately onto the suture ring of the prosthetic heart valve, then all sutures were straightened, and the prosthetic valve was pushed under the annulus to confirm that it was in the correct place and the prosthetic valve was not obstructed. Thorough flushing of the aorta and left ventricle, filling the aorta and left ventricle with saline. The aortic incision was closed with two consecutive sutures using 5–0 sutures, and after venting the left heart and ascending aorta, the ascending aortic blocking clamp was opened. If not automatically resuscitated, resuscitation with electric defibrillation is used. After resuscitation, the heart was left to beat without load for a period, and then the superior and inferior vena cava blocking bands were opened to enter parallel circulation. After a period of assisted circulation, the cardiopulmonary bypass was turned off.

### 2.4. Post-operative monitoring

Patients receive an intravenous infusion of amiodarone at a total dose of 1200 mg for 24 h postoperatively (amiodarone is discontinued when the voluntary heart rate falls below 70 beats per minute). Then oral amiodarone was administered to prevent the recurrence of AF. Regular monitoring of thyroid function was made during drug administration. The patient will continue to take oral amiodarone for 3–6 months and discontinue the drug depending on the results of the review. When pericardial/mediastinal drainage was reduced, heparin (10–20 mg, q6 h) was given to prevent thrombosis. Heparin was discontinued when the international normalized ratio (INR) is between 1.8 and 2.5. Warfarin anticoagulation continues for 6 months in patients with bioprosthetic valve replacement and whole life in patients with mechanical valve replacement. A rate-responsive pacemaker will be implanted if there is a III° atrioventricular block (AVB) that does not recover 7–10 days after the procedure.

### 2.5. Follow-Up

All patients were followed up at discharge and 6,12 and 18 months. The test items were 12-lead ECG, 24 h Holter monitoring, and echocardiography. The criteria for successful conversion and maintenance of sinus rate were defined as a clear sinus rate on the 12-lead ECG and the absence of AF on the 24 h Holter monitor. Atrial arrhythmia recurrence was defined as any documented episode of AF, atrial flutter, or atrial tachycardia >30 s after a 3-month gap period. Follow-up and treatment are performed by physicians independent of the principal investigator.

### 2.6. Statistical analyses

Statistical analyses were performed using IBM® SPSS® version 27.0 (IBM Corporation, Armonk, NY, United States). Categorical data are expressed as numbers (percentages) and compared using the chi-square test or Fisher's exact test. The normal distribution of continuous variables was assessed by the Kolmogorov-Smirnov test. Depending on whether the data were normally distributed, continuous data were expressed as mean ± standard deviation or median [interquartile ranges (IQRs)]. Student's *t*-test or Mann-Whitney *U*-test was used to compare these data depending on whether the data were normally distributed or not. A Cox proportional hazards regression model was used to adjust for confounding factors, variables with *P* < 0.05 in the univariate analysis were included in the multivariate regression model. All tests were two-tailed tests. The significance level was set at 0.05, with a 95% confidence interval.

## 3. Results

### 3.1. Patient characteristics

A total of 108 consecutive patients were enrolled in the study. There were 64 women (50%), and the median (IQR) durations of symptoms were 2 (0.9–5) years. The median (IQR) age was 59 (55–65) years, and most patients (88/108) had New York Heart Association (NYHA) functional class ≥ III. The two groups did not differ significantly in body mass index, blood pressure, previous medical history, routine blood test, or hepatic/renal functions. The baseline clinical characteristics of the study population are shown in Table 1. Compared with patients in the no Cox-maze group, those in the Cox-maze group had less cardio-thoracic ratio (63.05 ± 4.87 vs. 66.20 ± 6.55, *P* = 0.015).

**Table 1:** Baseline characteristics between two groups.

Baseline characteristics	Cox-maze (*N* = 58) %	No Cox-maze (*N* = 50) %	*P*-Value
Age (year), median (IQR)	60 (55,68)	60 (54,66)	0.910
Duration of symptoms (year), median (IQR)	3 (2,5)	1 (0.35,5)	0.254
Gender (Females), *n* (%)	36 (62.1)	28 (56)	0.522
BMI (kg/m^2^), mean ± SD	22.71 ± 3.12	22.59 ± 3.39	0.859
DBP (mmHg), mean ± SD	79.94 ± 15.75	71.29 ± 12.52	0.441
SBP (mmHg), mean ± SD	114.08 ± 22.91	112.78 ± 16.21	0.744
NYHA functional class III/IV, *n* (%)	50 (86)	38 (76)	0.173
Long-standing persistent AF, *n* (%)	38 (65)	29 (58)	0.422
Hypertension, *n* (%)	9 (15.5)	6 (12)	0.657
Coronary heart disease, *n* (%)	4 (6.9)	0 (0)	0.122
Diabetes mellitus, *n* (%)	3 (5.2)	1 (2)	0.719
Hyperlipidemia, *n* (%)	0 (0)	0 (0)	1
Cerebral hemorrhage, *n* (%)	0 (0)	0 (0)	1
Cerebral infarction, *n* (%)	10 (17.2)	5 (10)	0.278
Peripheral vascular disease, *n* (%)	0 (0)	1 (2)	0.463
Carotid/aortic plaque, *n* (%)	1 (1.7)	2 (4)	0.896
Hemorrhage, *n* (%)	2 (3.4)	0 (0)	0.498
Drug/alcohol addiction, *n* (%)	0 (0)	1 (2)	0.463
Abnormal thyroid function, *n* (%)	1 (1.7)	0 (0)	1
RBC (10^12^/L), mean ± SD	4.52 ± 0.11	4.41 ± 0.11	0.522
HGB (g/L), mean ± SD	129.9 ± 5.37	155.1 ± 22.93	0.349
WBC (10^9^/L), mean ± SD	5.87 ± 0.27	5.50 ± 0.30	0.378
GRAN (%), mean ± SD	60.07 ± 1.40	60.07 ± 1.53	0.999
PLT (10^9^/L), mean ± SD	197.1 ± 10.86	182.4 ± 9.04	0.291
TBIL (μ mol/L), mean ± SD	16.57 ± 1.65	14.71 ± 0.96	0.313
AST (U/L), mean ± SD	24.93 ± 2.20	30.06 ± 3.08	0.200
ALT (U/L), mean ± SD	26.55 ± 2.45	29.04 ± 6.36	0.739
Creatinine (μ mol/L), mean ± SD	75.81 ± 3.18	76.04 ± 3.45	0.962
BUN (μ mol/L), mean ± SD	6.59 ± 0.34	7.44 ± 0.42	0.132
INR, mean ± SD	1.42 ± 0.101	1.21 ± 0.07	0.110
PT(s), mean ± SD	15.63 ± 1.03	14.02 ± 0.75	0.219
CHA2DS2-VASc, mean ± SD	1.35 ± 0.27	1.62 ± 0.62	0.672
Cardio-thoracic ratio (%)	63.05 ± 4.87	66.20 ± 6.55	0.015
Preoperative LA (mm), mean ± SD	48.83 ± 6.52	49.78 ± 9.20	0.547
Preoperative RA (mm), mean ± SD	35.46 ± 4.93	38.46 ± 6.51	0.009
Preoperative LV (mm), mean ± SD	50.75 ± 7.49	53.23 ± 8.50	0.118
Preoperative RV (mm), mean ± SD	33.55 ± 3.62	34.95 ± 5.09	0.107
Preoperative EF (%), mean ± SD	60.33 ± 9.32	59.84 ± 9.64	0.795
LVEF > 50%, *n* (%)	47 (81)	43 (86)	0.363
Peak aortic jet velocity(m/s), mean ± SD	3.90 ± 0.20	3.70 ± 0.13	0.005
Mean gradient (mm Hg), mean ± SD	35.02 ± 3.26	38.28 ± 1.86	0.028
Aortic valve area (cm^2^), mean ± SD	1.10 ± 0.02	1.09 ± 0.08	0.171
Isolated AS, *n* (%)	28 (48)	22 (44)	0.657
Concomitant MVD, *n* (%)	30 (52)	28 (56)	0.657
Preoperative Warfarin, *n* (%)	58 (100)	50 (100)	1
Preoperative AAD, *n* (%)	58 (100)	50 (100)	1

BMI, body mass index; DBP, diastolic blood pressure; SBP, systolic blood pressure; NYHA, New York heart association; LA, left atrium; RA, right atrium; LV, left ventricle; RV, right ventricle; EF, ejection fractions; HGB, hemoglobin; WBC, white blood cells; GRAN, neutrophilic granulocyte; PLT, platelets; TBIL, total bilirubin; AST, aspartate aminotransferase; ALT, alanine aminotransferase; CR, Creatinine; BUN, blood urea nitrogen; INR, international normalized ratio; PT, prothrombin time; RBC, red blood cells; AS, aortic stenosis; MVD, mitral valve disease; AAD, anti-arrhythmic drugs; SD, standard deviation; IQR, interquartile range.

Table 1 also provides information on the pre-operative echocardiographic data of patients in the Cox-maze and no Cox-maze groups. Patients in the Cox-maze group had significantly smaller pre-operative right atrial diameters (35.46 ± 4.93 vs. 38.46 ± 6.51, *P* = 0.009) and slightly smaller left ventricular end-diastolic diameters (50.75 ± 7.49 vs. 53.23 ± 8.50, *P* = 0.118) than those in the no Cox-maze group, but the latter's difference was not statistically significant.

### 3.2. Comparison of perioperative characteristics between two groups

Aortic valve replacement was performed in all patients, combined with the Cox-maze procedure in 58 (53.70%) patients, and not combined with the Cox-maze procedure in 50 (46.29%) patients. 64 (59%) patients underwent surgery with a mechanical valve and 44 (41%) with a biological valve. Most patients (79/108) underwent left atrial appendectomy. We found 8 (7.4%) patients with left atrial thrombosis during the surgery. Patients in the Cox-maze group had longer cardiopulmonary bypass time (126 [109.5–142] vs. 92.00 [71–105] min, *P* < 0.001) and aortic cross-clamp time (86 [79–97.5] vs. 61.00 [42–72] min, *P* < 0.001). Nevertheless, the length of postoperative hospital stays (9 [8–12] vs. 9 [7–13], *P* = 0.489), and the frequency of serious complications did not differ across the groups (Table 2).

**Table 2:** Comparison of perioperative characteristics between two groups.

Post-operative data	Cox-maze (*N* = 58) (%)	No Cox-maze (*N* = 50) (%)	*P*-Value
Mechanical prosthesis, *n* (%)	34 (58)	30 (51)	0.884
Left atrial thrombosis, *n* (%)	3 (5)	5 (10)	0.557
CPB time (min), median (IQR)	126 (109.5,142)	92 (71,105)	<0.001
Aortic clamp time (min), median (IQR)	86 (79,97.5)	61 (42,72)	<0.001
Length of stay (day), median (IQR)	9 (8,12)	9 (7,13)	0.489
Perioperative stroke, *n* (%)	0	1	0.463
Perioperative death, *n* (%)	0	0	1
Pacemaker implantation, *n* (%)	0	1	1
CRRT, *n* (%)	1	0	1
IABP, *n* (%)	1	0	1
Re-intubation, *n* (%)	0	0	1
Poor healing of surgical wound, *n* (%)	0	1	0.463
Pericardial effusion, *n* (%)	0	1	0.463

CPB, cardiopulmonary bypass; CRRT, continuous renal replacement therapy; IABP, intra-aortic balloon pump; IQR, interquartile range.

During the perioperative period, no death or stroke events occurred in both two groups, but one patient received a pacemaker implanted because of III° atrioventricular block (AVB); one patient received continuous renal replacement therapy (CRRT) because of renal failure; one patient received intra-aortic balloon pump because of low cardiac output syndrome, and one re-hospitalization has occurred because of poor healing of the surgical wound.

### 3.3. Follow-Up

The median (IQR) follow-up was 198 (175–311) days. There were three deaths in the no Cox-maze group during follow-up. One patient died due to heart failure 60 days after the discharge, one due to malignancy 311 days after discharge, and one died 145 days after discharge, but his kinship was reluctant to reveal the cause of his death.

Patients in the Cox-maze group saw a decreased mid-term AF recurrence compared to those in the no Cox-maze group (*P* = 0.004). Compared with the patients in the Cox-maze group, the patients in no Cox-maze group had higher total bilirubin (18.31 ± 1.45 vs.12.82 ± 0.64, *P* = 0.001), aspartate aminotransferase (33.83 ± 2.36 vs. 27.71 ± 1.19, *P* = 0.014), and larger left atrial diameters (44.4 ± 1.89 vs. 40.11 ± 1.10, *P* = 0.044), the differences are statistically significant (Table 3). In the subgroup analysis of the relationship between gender and surgical prognosis outcomes, we found no effect of gender on all-cause mortality and postoperative AF recurrence.

**Table 3:** Comparison of characteristics in follow-up between two groups.

Follow-up data	Cox-maze (*N* = 58)	No Cox-maze (*N* = 50)	*P*-Value
RBC (10^12^/L), mean ± SD	4.45 ± 0.09	4.434 ± 0.09	0.902
HGB (g/L), mean ± SD	131.8 ± 2.50	130 ± 2.52	0.687
WBC (10^9^/L), mean ± SD	6.54 ± 0.29	5.639 ± 0.25	0.079
GRAN (%), mean ± SD	65.13 ± 1.46	65.62 ± 2.26	0.858
PLT (10^9^/L), mean ± SD	203.2 ± 7.44	172.2 ± 18.05	0.063
TBIL (μ mol/L), mean ± SD	12.82 ± 0.64	18.31 ± 1.45	<0.001
AST (U/L), mean ± SD	27.71 ± 1.19	33.83 ± 2.36	0.014
ALT (U/L), mean ± SD	22.83 ± 1.72	28.21 ± 2.74	0.106
CR (μ mol/L), mean ± SD	73.24 ± 2.36	68.46 ± 3.71	0.290
BUN (μ mol/L), mean ± SD	5.87 ± 0.22	5.906 ± 0.43	0.946
INR, mean ± SD	1.86 ± 0.08	2.02 ± 0.18	0.377
PT(s), mean ± SD	20.74 ± 0.78	22.04 ± 1.71	0.431
Postoperative LA (mm), mean ± SD	40.11 ± 1.10	44.42 ± 1.89	0.044
Postoperative RA (mm), mean ± SD	33.02 ± 0.59	35.33 ± 1.19	0.059
Postoperative LV (mm), mean ± SD	45.56 ± 1.17	45.33 ± 1.48	0.915
Postoperative RV (mm), mean ± SD	30.51 ± 1.12	32.78 ± 0.90	0.232
Postoperative EF (%), mean ± SD	63.16 ± 1.52	62.06 ± 2.83	0.715

RBC, red blood cells; HGB, hemoglobin; WBC, white blood cells; GRAN, neutrophilic granulocyte; PLT, platelets; TBIL, total bilirubin; AST, aspartate aminotransferase; ALT, alanine aminotransferase; CR, Creatinine; BUN, blood urea nitrogen; INR, international normalized ratio; PT, prothrombin time; LA, left atrium; RA, right atrium; LV, left ventricle; RV, right ventricle; EF, ejection fractions; SD, standard deviation.

**Figure 1:**
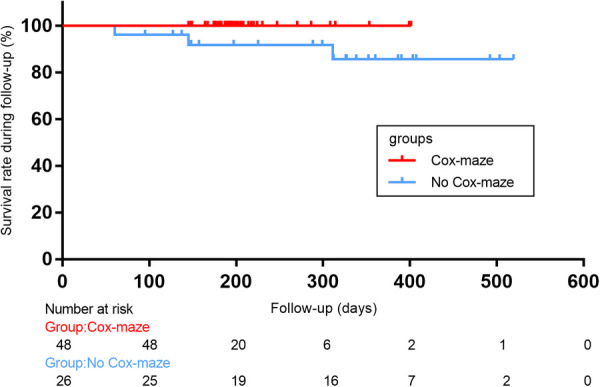
Kaplan-Meier overall survival curve after concomitant Cox-maze IV procedure with surgical aortic valve replacement in patients with CAVD and AF. Log-rank statistic 3.915, *p* = 0.046.

Freedom from all-cause mortality following aortic valve replacement at 12 months was 100% in the Cox-maze group during the follow-up period, compared to 89%, respectively, in the no Cox-maze group. Patients in the no Cox-maze group had a poorer survival rate than those in the Cox-maze group, according to the Kaplan-Meier survival curves (*P* = 0.048, Figure 1).

### 3.4. AF recurrence outcome

**Figure 2:**
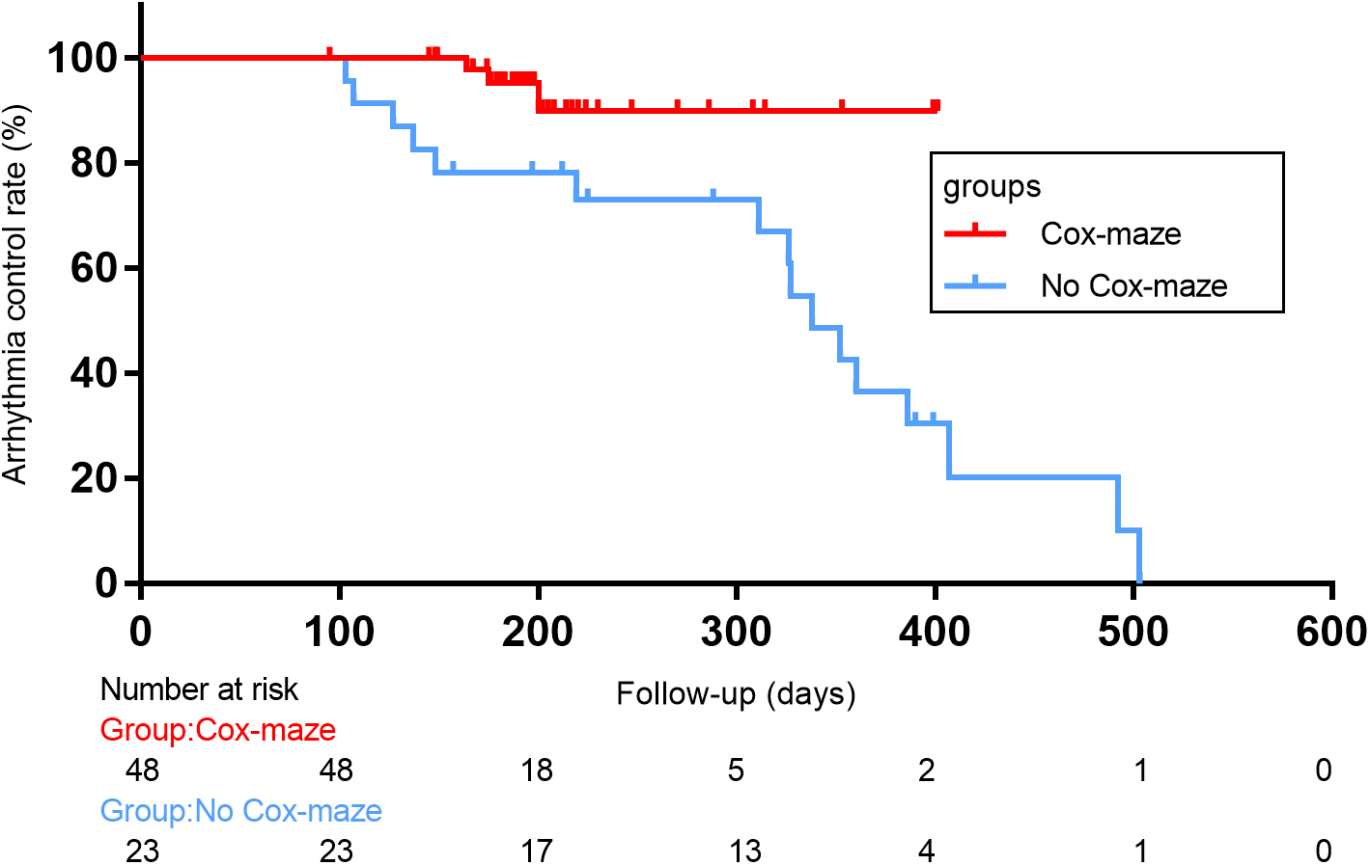
Arrhythmia control rates (including patients with successful AADs conversion)after concomitant Cox-maze IV procedure with surgical aortic valve replacement in patients with CAVD and AF. Log-rank statistic 6.281, *p* = 0.012.

**Figure 3:**
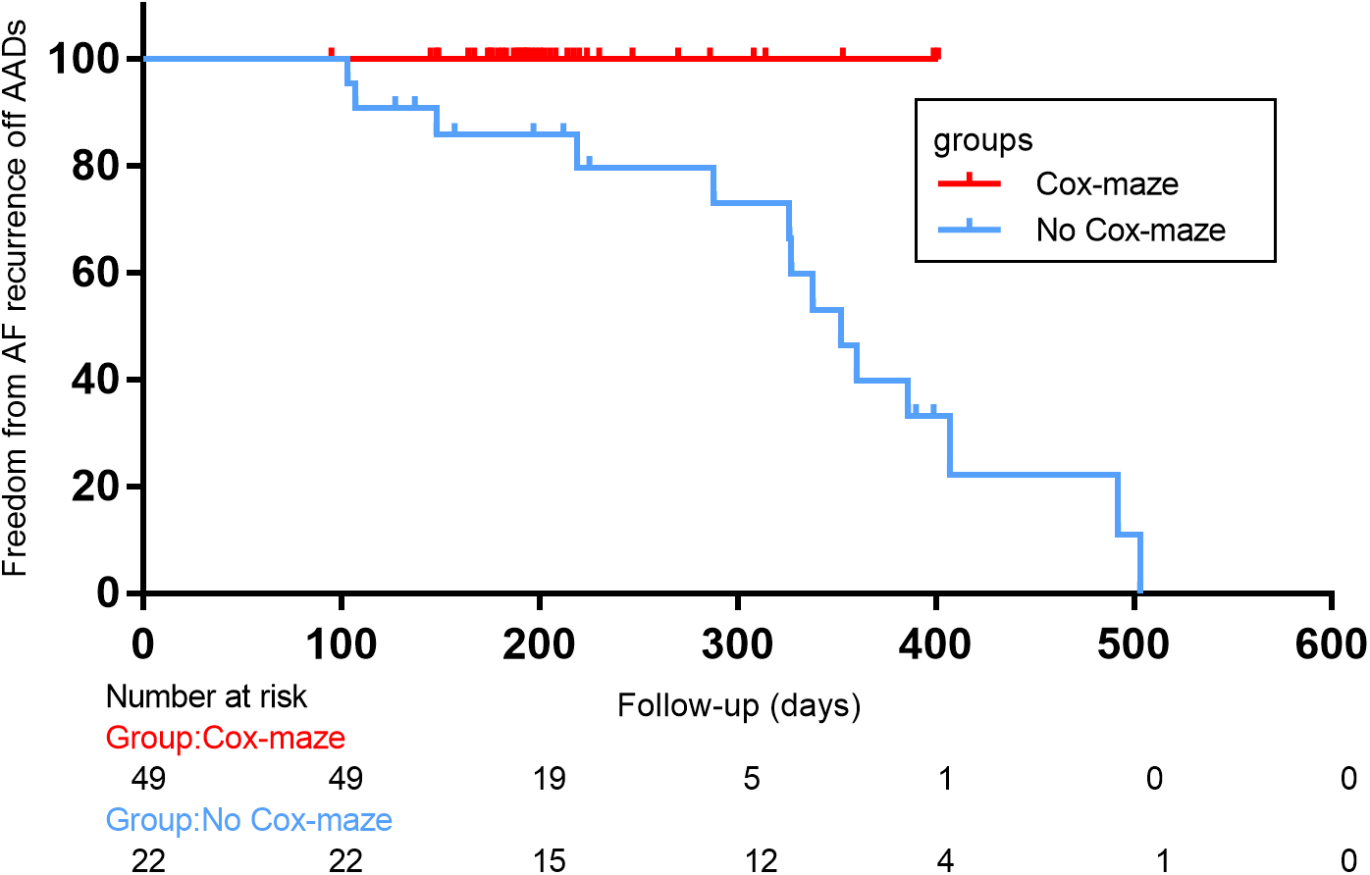
Freedom from atrial fibrillation (AF) recurrence off antiarrhythmic drugs (AADs) after concomitant Cox-maze IV procedure with surgical aortic valve replacement in patients with CAVD and AF. Log-rank statistic 8.422, *p* = 0.004.

In total, there were 14 occurrences of recurrent AF, including 0 cases in the Cox-maze group and 14 cases in the no Cox-maze group. In the Cox-maze group, freedom from AF recurrence off antiarrhythmic drugs (AADs) was 100% at 12 months, and the arrhythmia control rates (including patients with successful AADs conversion) were 95%, respectively. As shown in Figures 2,3, the Kaplan–Meier survival curve showed patients in the no Cox-maze group had a lower rate of freedom from AF recurrence off AADs and a better arrhythmia control than those in the Cox-maze group (*P* = 0.003 and *P* = 0.012, respectively). In the group of all patients who presented with postoperative AF recurrence, 21.4% of patients (3/14) had recurrence between 100 and 200 days after surgery,14.2% of patients (2/14) had recurrence between 200 and 300 days after surgery, and 42.8% of patients (6/14) had recurrence between 300 and 400 days after surgery, 21.4% of patients (3/14) had recurrence longer than 400 days after surgery. After adjusting for confounding factors, the multivariate Cox proportional hazards regression analysis revealed that pre-operatively increased systolic blood pressure (SBP) (hazard ratio, 1.096; 95% CI, 1.004–1.196; *P* = 0.04) and post-operatively increased right atrium diameters (RADs) (hazard ratio, 1.755; 95% CI, 1.182–2.604; *P* = 0.005) were associated with AF recurrence (Table 4).

**Table 4:** Predictors of AF recurrence following AVR.

Variables	Univariate model		Multivariate model	
	Hazard ratio (95% CI)	*P*-value	Hazard ratio (95% CI)	*P*-value
Age	1.094 (1.004–1.191)	0.04	1.039 (0.898–1.203)	0.608
Female gender	0.684 (0.23–2.037)	0.495		
Average BMI	0.935 (0.995–1.064)	0.366		
SBP	1.029 (0.995–1.064)	0.099	1.096 (1.004–1.196)	0.04
DBP	1.054 (0.998–1.113)	0.06		
Cerebral infarction	0.239 (0.048–1.201)	0.082		
Hypertension	2.797 (0.878–8.91)	0.082		
Preoperative EF (%)	0.983 (0.917–1.053)	0.62		
Preoperative INR	0.893 (0.306–2.612)	0.837		
CHA2DS2-VASc	0.981 (0.642–1.497)	0.928		
Cardiothoracic ratio	0.958 (0.658–1.395)	0.823		
Preoperative LA	1.124 (1.048–1.205)	0.001	0.97 (0.842–1.117)	0.67
Preoperative RA	1.275 (1.116–1.455)	0.001	1.755 (1.182–2.604)	0.005
Preoperative LV	1.044 (0.973–1.119)	0.228		
Preoperative RV	1.189 (1.028–1.376)	0.02	0.82 (0.636–1.057)	0.125
Postoperative LA	1.156 (1.027–1.303)	0.017	1.144 (0.928–1.409)	0.207
Postoperative RA	1.259 (1.078–1.47)	0.004	0.688 (0.446–1.062)	0.091
Postoperative LV	1.019 (0.936–1.109)	0.663		
Postoperative RV	1.361 (1.109–1.669)	0.003	1.102 (0.687–1.77)	0.686

BMI, body mass index; DBP, diastolic blood pressure; SBP, systolic blood pressure; LA, left atrium; RA, right atrium; LV, left ventricle; RV, right ventricle; EF, ejection fractions; CI, confidence interval.

**Table 5:** Correlation between all-cause death, AF recurrence and gender of patients.

Item		Male	Female	*P*-Value
All-cause death	YES	2	1	0.375
	NO	29	42	
AF recurrence	YES	5	9	0.856
	NO	23	37	

## 4. Discussion

In this study, we reported outcomes of a randomized controlled clinical trial of Cox-maze surgery in calcific aortic valve disease (CAVD) patients combined with AF. We found that compared with patients in the no Cox-maze group, those in the Cox-Maze group experienced the same hospital stay time and the possibility of perioperative complications. Meanwhile, the patients in the Cox-maze group had better cardiac remodeling changes, and a higher possibility of relief from AF during the follow-up period. Our results agree with previous publications. Churlya et al. (7) conducted a large retrospective clinical study and found that concomitant Cox-maze surgery and AVR is safe but with no advantage in postoperative survival. The authors discuss that inclusion of patients with mitral valve replacement may be a source of confusion and explain controversial results (6). Nevertheless, despite the small sample size of our study, we reported that Cox-maze surgery improves the postoperative survival. Meanwhile, a similar study conducted by Malaisrie et al. in 2012 also suggested that AVR combined with Cox-maze surgery slightly increased operative time but did not significantly increase postoperative complications and mortality (20). Combined with the above findings, we believe that the question of whether Cox-maze surgery is beneficial to patients' postoperative survival remains controversial and deserves continued discussion in future studies.

There is a high incidence of AF in heart valve disease. A total of 80% of patients with mitral stenosis and systemic embolism suffer from AF, and it is estimated that AF is responsible for 25% of deaths from systemic embolism when surgery and anticoagulation are not available (21). However, to date, there are fewer studies on the relationship between CAVD and AF. We can only know from the clinical database (Loire Valley Atrial Fibrillation Project) that aortic valve stenosis is present in 32% of patients who suffer from AF combined with valvular disease (13, 22). The previous study has found that even in asymptomatic or minimally symptomatic patients with severe AS, AF is a strong predictor of mortality. After aortic valve replacement (AVR), AF is still associated with a lower survival rate than sinus rhythm (23). But Hongju Z et al. hold the opposite view. They reveal that other factors than AF explained apparent differences in outcomes when compared to sinus rhythm, including concomitant cardiac abnormalities and postponement of AVR due to AF-related cardiac symptoms (16). In the present study, patients' baseline characteristics were equal among the two groups. In this condition, we treated AF using the Cox-maze IV procedure, which resulted in a significantly better mid-term prognosis for the AVR procedure compared to the no Cox-maze group. Our conclusions support the views of Levy F et al. (23) and Kubala M et al. (13). Different etiologies of valvular disease might account for this disparity. In previous studies, they did not, however, distinguish between rheumatic heart disease and CAVD in included patients. Rheumatic heart disease is the leading cause of valve disease in developing countries, but in western countries, AS has now become the most common valvulopathy. There is a pathological difference between these two diseases: if the rheumatism is repeatedly active, and the exudate is not easily absorbed, it can form a redundancy, making the valve fibrotic and adhesive, which eventually leads to valve stenosis and insufficient. As for CAVD, however, the mechanism involved in its pathogenesis of it remains controversial: lipoprotein deposition, inflammation, and osteogenic transition of cardiac valve interstitial cells may play a role in its pathogenesis (24). Notably, there are differences between males and females in the pathogenesis of CAVD. This leads to the result of women appearing to have lower levels of calcification but still having poorer outcomes after AVR and higher rates of atrial fibrillation and stroke (25, 26). Research has shown that compared to men, women have fewer calcified valves but more fibrosis (27). The mechanisms may include the aldosterone/mineralocorticoid receptor pathway and sex-dependent expression of neutrophil gelatinase-associated lipocalin (28, 29). Previous studies have also shown women with AF may have more side effects on AAD, higher stroke risk, more disabling strokes, and higher cardiovascular mortality. But interestingly, in research about Cox-maze surgery, no significant sex differences could be found in the outcome freedom from AF without AAD treatment at last follow-up (30, 31). This is consistent with the findings of our study (Table 5).

Few studies are reporting a direct relationship between AF and CAVD, however, we can still find clues from independent studies. According to Dai W et al.'s research, HOTAIR, which acts as a ceRNA by sponging miR-613, is a significant factor in the remolding of Cx43 in AF (32). Carrion K et al. demonstrated that HOTAIR is mechanoresponsive and repressed by WNT b-CATENIN signaling. This is the role of HOTAIR in the calcification of human aortic valve interstitial cells (33). The above study found a protective effect of the long non-coding RNA HOTAIR for both AF and CAVD. These results suggest that there may be a molecular mechanism associated with AF and CAVD. This also explains the high conversion rate of Cox-maze surgery in patients with CAVD combined with AF (100% in the present study vs. 60%–70% in the previous study): this may be due to the removal of calcification from the aortic valve, which mitigates the risk factors for the development of AF (34–40).

Previous studies suggested that the major independent predictors of ablation failure are atrial fibrillation/flutter at discharge, preoperative right atrial diameter, hypertension, diabetes, and smoking (41). However, there is limited evidence from patients with CAVD combined with AF. Our study supports part of the conclusions of previous studies: the multivariate Cox regression analysis revealed that SBP (hazard ratio, 1.096; 95% CI, 1.004–1.196; *P* = 0.04) and pre-operative RADs (hazard ratio, 1.755; 95% CI, 1.182–2.604; *P* = 0.005) were linked to AF recurrence. Because fewer of our included patients had a history of diabetes and smoking, therefore, the present study failed to conclude the predictive value of these two variables for the recurrence of AF.

Similar to the studies reported in hypertrophic obstructive cardiomyopathy by Meng Y et al. (42), the univariate Cox proportional hazards regression analysis revealed that large post-operative LADs (hazard ratio, 1.156; 95% CI, 1.027–1.303; *P* = 0.017) can predict the recurrence of atrial filiation after Cox-maze surgery. However, we did not obtain this outcome in the subsequent multi-factor regression analysis ([hazard ratio, 1.144; 95% CI, 0.928–1.409; *P* = 0.207] in our research vs. [hazard ratio, 1.099; 95% CI, 1.024–1.409; *P* = 0.009] in Meng's research). The following explanations could explain this difference: there was continuous variable collinearity (LADs, RADs, and left ventricular end-diastolic diameters [LVEDDs]) in the data for which we performed multifactorial regression analysis, which leads to disparity in results. In conclusion, our findings indicate that the AF recurrence rate in patients who underwent the Cox-maze IV procedure was lower than in patients who did not receive the procedure, confirming that the Cox-maze IV procedure should be actively performed in such patients to maintain post-operative SR.

## 5. Study limitations

There are some limitations to this study. This was a single-center prospective control study with a relatively small sample size and insufficient follow-up data. In the present study, although G power calculation revealed a sufficient sample size, this only means that the minimum standards are met. As a result, performing a meaningful and extensive statistical analysis is difficult. Since few previous studies have separately examined the surgical treatment of CAVD combined with AF, our total surgical volume was relatively high. Previous studies on the surgical treatment of patients with heart valve disease and AF were mostly retrospective observational studies, so the prospective character of our study provides an opportunity to enhance its importance. Another limitation is that, due to the surgeon's experience and the patient's conditions, we used a variety of ablation equipment and valve prostheses, including Medtronic and AtriCure, while the present study lacked relevant subgroup analysis. Large-scale prospective studies are required in the future to confirm the efficacy and safety of various ablation strategies, equipment, and valve prosthesis. Moreover, these study's findings might be conditioned by the cohort's peculiarities. For example, women represent more than 50% of the two groups and yet the mean age is around 60 years old, normal range of BMI. Therefore, these factors may interfere with the external validity of the results of this study.

## 6. Conclusion

In conclusion, our study demonstrated that in patients with CAVD and AF, the Cox-maze IV technique in conjunction with aortic valve replacement increased mid-term survival and decreased mid-term AF recurrence. In patients with CAVD and AF, the concurrent Cox-maze IV procedure is linked to a decreased AF recurrence rate.

## Data Availability

The raw data supporting the conclusions of this article will be made available by the authors, without undue reservation.
